# Protective role of beta-blockers in chemotherapy-induced cardiotoxicity—a systematic review and meta-analysis of carvedilol

**DOI:** 10.1007/s10741-018-9755-3

**Published:** 2018-12-06

**Authors:** Shan Huang, Qin Zhao, Zhi-gang Yang, Kai-yue Diao, Yong He, Ke Shi, Meng-ting Shen, Hang Fu, Ying-kun Guo

**Affiliations:** 1 Department of Radiology, Key Laboratory of Birth Defects and Related Diseases of Women and Children of Ministry of Education, West China Second University Hospital, Sichuan University, Chengdu, China; 2 Department of Radiology, West China Hospital, Sichuan University, 37# Guo Xue Xiang, Chengdu, 610041 Sichuan China; 3 Department of Cardiology, West China Hospital, Sichuan University, 37# Guo Xue Xiang, Chengdu, 610041 China

**Keywords:** Beta-blockers, Carvedilol, Chemotherapy, Anthracyclines, Cardiotoxicity

## Abstract

Some randomized controlled trials (RCTs) have tested the efficacy of beta-blockers as prophylactic agents on cancer therapy-induced cardiotoxicity; however, the quality of this evidence remains undetermined. This systematic review and meta-analysis study aims to evaluate the prophylactic effects of beta-blockers, especially carvedilol, on chemotherapy-induced cardiotoxicity. RCTs were identified by searching the MEDLINE (PubMed), Embase (OvidSP), Cochrane CENTRAL (OvidSP), etc., until December 2017. Inclusion criteria were randomized clinical trial and adult cancer patients started beta-blockers before chemotherapy. We evaluated the mean differences (MD) by fixed- or random-effects model and the odds ratio by Peto’s method. Primary outcome was the left ventricular ejection fraction (LVEF) of patients after chemotherapy, and secondary outcomes were all-cause mortality, clinically overt cardiotoxicity, and other echocardiographic measurements. In total, we included six RCTs that used carvedilol as a prophylactic agent in patients receiving chemotherapy. The LVEF was not significantly distinct between those using carvedilol and placebo after chemotherapy (MD, 1.74; 95% confidence interval (CI), − 0.18 to 3.66; *P* = 0.08). The incidence of clinically overt cardiotoxicity was lower in the carvedilol group compared with the control group (Peto OR, 0.42; 95% CI, 0.20–0.89; *P* = 0.02). Furthermore, after chemotherapy, the LV end-diastolic diameter did not increase in the carvedilol group compared with the placebo group (MD, − 1.41; 95% CI, − 2.32 to − 0.50; *P* = 0.002). The prophylactic use of carvedilol exerted no impact on the early asymptomatic LVEF decrease but seemed to attenuate the frequency of clinically overt cardiotoxicity and prevent ventricular remodeling.

**Electronic supplementary material:**

The online version of this article (10.1007/s10741-018-9755-3) contains supplementary material, which is available to authorized users.

## Introduction

Recent advancements in cancer treatment have remarkably improved the overall survival of patients with cancer [1]. However, chemotherapy-induced cardiotoxicity has been related to unfavorable prognosis in cancer survivors [2, 3], which has put oncologists into a therapeutic dilemma to maintain a balance between the antitumor efficacy and cardiac injury.

Heterogeneous factors contributing to chemotherapy-induced cardiotoxicity are cumulative anthracycline doses [4], adjunct therapy with trastuzumab [5, 6], prior radiotherapy [7], preexisting cardiovascular risks (e.g., diabetes mellitus, hypertension, and dyslipidemia) [8, 9], and age [10]. Multiple mechanisms are associated with anthracycline-induced cardiotoxicity; however, the most extensively accepted mechanism seems to be the generation of oxygen free radicals, which cause cardiomyocyte damage and apoptosis [11].

Lately, various efforts have been made to attenuate chemotherapy-induced cardiotoxicity, including early detection of subclinical toxicity, development of derivatives [12], and the prophylactic use of cardioprotectants. The effects of cardioprotective beta-blockers have been tested in multiple animal experiments and clinically randomized controlled trials (RCTs). The primary mechanism associated with beta-blockers is reduction of oxidative stress and apoptosis [13, 14]. Carvedilol is a nonselective β and α1 adrenergic antagonist and has been proven to be cardioprotective not only through the intrinsic β-blocking effect (attenuating the sympathetic activity and cardiac remodeling) [15] but also the additional antioxidant and anti-inflammation properties compared with other beta-blockers [16, 17]. In particular, carvedilol can protect the myocardium without interfering with the antineoplastic efficacy of anthracyclines [18]. Although several beta-blockers have been tested through clinical RCTs, the number of enrolled patients was limited, and the results remained debatable. This study aims to evaluate the prophylactic effects of beta-blockers, especially carvedilol, on chemotherapy-induced cardiotoxicity by systematic review and meta-analysis.

## Methods

This systematic review was conducted in accordance with the Preferred Reporting Items for Systematic Reviews and Meta-Analyses (PRISMA) statement [19]. This study was prospectively registered with the PROSPERO database of systematic reviews (registration number: CRD42018086747).

### Search strategy

We systematically searched MEDLINE (PubMed), Embase (Ovid SP), Cochrane CENTRAL (Ovid SP), and the World Health Organization International Clinical Trials Registry Platform, as well as two Chinese medical databases, CNKI and WANFANG, until December 2017. In addition, a follow-up search was conducted for ongoing trials. The search strategy comprised a combination of Medical Subject Headings terms and free-text terms, primarily including “carvedilol,” “beta-blockers,” “anthracycline,” “chemotherapy,” “cardiotoxicity,” “cardiomyopathy,” and “heart failure.” Furthermore, we reviewed the reference lists of relevant studies and review articles. Of note, we limited the search to human beings without any language restriction.

### Inclusion and exclusion criteria

The inclusion criteria were as follows: (a) patients with cancer aged > 18 years due to receive chemotherapy; (b) beta-blockers used against the cardiotoxic effect of chemotherapy in a prophylactic setting (started before chemotherapy); (c) prospective RCT study design; and (d) left ventricular ejection fraction (LVEF) assessed both at the baseline and post-chemotherapy. We excluded studies in which beta-blockers were used concomitantly with other cardioprotective agents, such as angiotensin-converting enzyme inhibitors.

### Data extraction and study quality assessment

Data extraction and study quality assessment were performed per the PRISMA statement [19]. For systematic review, the following data and information were extracted: study design, age, gender, sample size, type of cancer, cumulative anthracycline doses, beta-blockers, follow-up time, baseline LVEF, and outcomes. The primary outcome was LVEF after chemotherapy, and secondary outcomes were all-cause mortality, clinically overt cardiotoxicity, and other cardiac measurements (e.g., E/A ratio and LV chamber size).

The quality of studies was assessed per the Cochrane risk of bias tool [20]. The adequacy of blinding was ascertained by whether cardiac measurements were assessed by a third person blinded to patients’ information. Any disagreement was resolved by consensus. For key information that was not reported in the articles, we contacted the authors by e-mails.

### Statistical analysis

We performed the statistical analyses using Review Manager (V.5.3, Cochrane) and Stata 13.0 (Stata Corp LP, College Station, TX). Heterogeneity among studies was assessed using the *Q* test and *I*^2^ statistics (*I*^2^ > 50% suggested substantial heterogeneity). In the absence of considerable heterogeneity, the fixed effects model was selected to obtain the mean difference (MD) and 95% confidence interval (CI); else, the random effects model was used. For comparing the risk of adverse events and mortality in patients and control subjects, we used Peto’s one-step odds ratio method, which is the least biased and most potent method when event rates were low, per the Cochrane handbook [21]. We considered *P* < 0.05 as statistically significant. In addition, the Galbraith plot and sensitivity analysis were conducted to determine the primary source of heterogeneity and ascertain the stability of the statistical results. Publication bias was assessed by Egger’s test. Although we planned to perform subgroup analysis, trials were insufficient.

## Results

### Search results

**Fig. 1:**
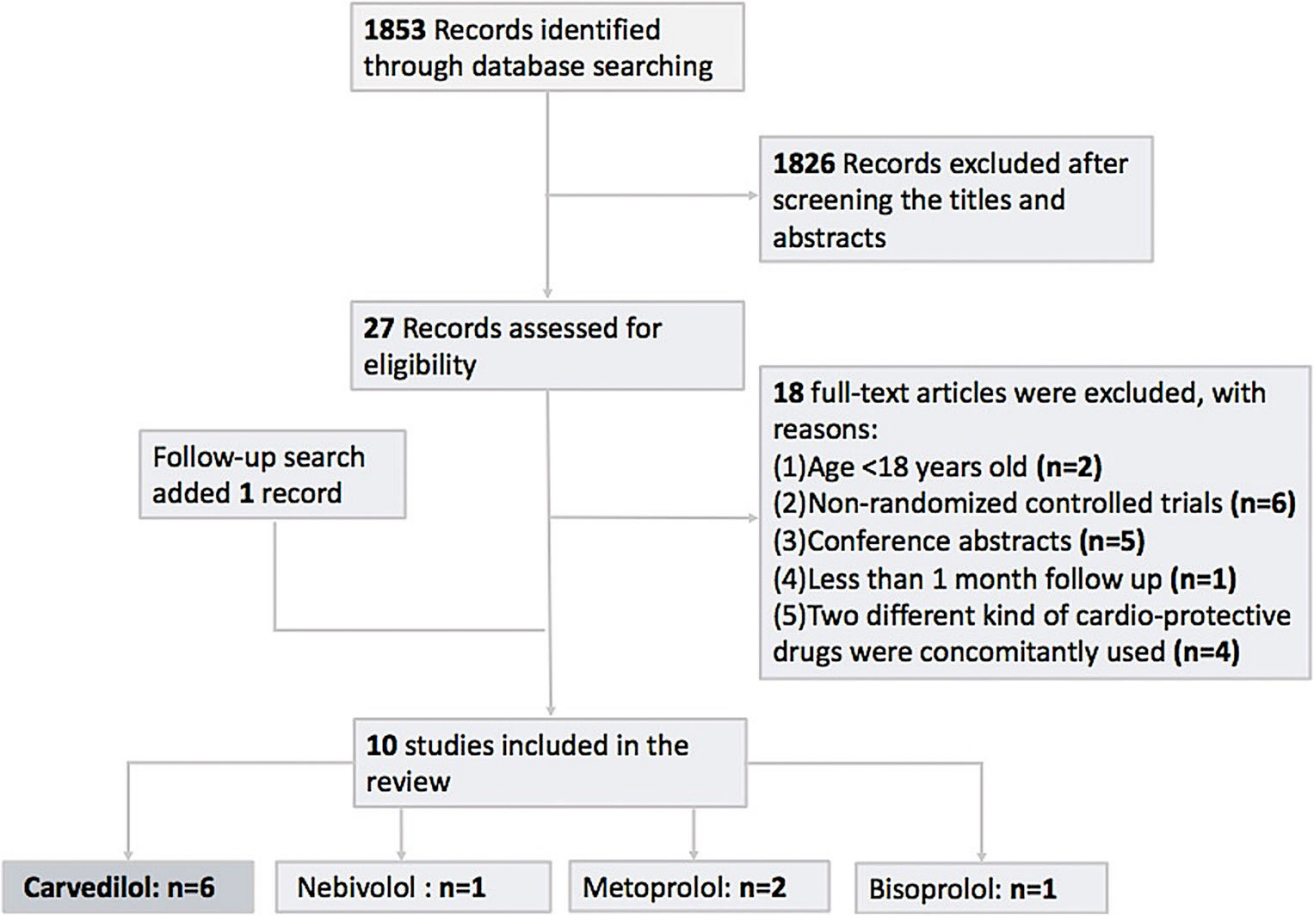
Flow chart of article screening process. This flow chart describes how the included studies were selected for the systematic review and meta-analysis. The follow-up search conducted 3 months later added one study

Initially, our comprehensive search yielded 1853 articles. We excluded duplicates and other 1628 articles after screening the titles and abstracts. We scrutinized the full texts of the remaining 27 studies, of which 17 were excluded for various reasons (Fig. 1). Overall, we included 10 studies (6 on carvedilol, 1 on nebivolol, 2 on metoprolol, and 1 on bisoprolol). We analyzed the statistical data from six studies on carvedilol.

### Study and patient characteristics

For 10 studies, 775 patients were included (male/female, 101/674; average age, 48.6 years) with various malignancies, including breast cancer, lymphoma (non-Hodgkin and Hodgkin), leukemia, and others, among which the rate of breast cancer was the highest. In fact, six studies exclusively included patients with breast cancer. All patients received anthracycline-containing chemotherapy. Patients received a relatively high cumulative anthracycline dose (> 550 mg/m^2^) in four studies and a relatively low dose in six studies. Patients in Pituskin’s study [22] received trastuzumab as an adjunct therapy. And all patients exhibited normal LVEF (> 50%) at the baseline with no history of heart failure symptoms or coronary condition. In addition, all patients started beta-blockers (carvedilol [*n* = 182], nebivolol [*n* = 27], bisoprolol [*n* = 31], and metoprolol [*n* = 74]) before the initiation or on the first day of chemotherapy and continued until the end of the study. The median follow-up duration was 6 months (range, 10–61 weeks). Each study comprised an age- and a sex-matched control group receiving placebo. The left ventricular function was assessed mostly by 2D echocardiogram, except two studies using cardiac magnetic resonance imaging [22, 23] (Table 1).

**Table 1:** Characteristics of studies using carvedilol

Author year	Drug dosage (mg q.d.)	Type of cancer	Sample size (female %)		Age, years		Cumulative anthracycline doses (mg/m^2^)		Baseline LVEF, %		FU (mon.)
			Exp.	Con.	Exp.	Con.	Exp.	Con.	Exp.	Con.	
N. K. (2006) [24]	12.5	BC, lymphoma, etc.	25 (88%)	25 (84%)	46.8 ± 14	49.0 ± 9.8	ADM 525.3 EPI 787.9	ADM 513.6 EPI 770.4	70.6 ± 8.0	69.7 ± 7.3	6
R. J. (2014) [25]	12.5	NHL, HD, ALL	27 (14.8%)	27 (33.3%)	43.89 ± 15.66	38.74 ± 18.36	267.36	252.65	63.19 ± 7.22	67.56 ± 5.98	6
A. E. (2014) [26]	12.5	BC	40 (100%)	40 (100%)	54.3 ± 9.3	52.9 ± 11.2	535.6	523.3	66.0 ± 6.1	65.0 ± 4.5	6
M. N. (2017) [27]	Average 6.71 ± 1.65	BC	46 (100%)	45 (100%)	47.57 ± 8.75	47.1 ± 12.17	359.91	348.56	58.72 ± 4.69	61.13 ± 4.97	6
M. A. (2018) [28]	Maximal 18.5 ± 17.6	BC	96 (100%)	96 (100%)	50.8 ± 10.10	52.9 ± 9.05	240	240	64.8 ± 4.7	65.2 ± 3.6	6
R. S. (2011) [29]	12.5	BC, lymphoma	22 (68%)	22 (64%)	45.70 ± 14.16	43.50 ± 15.27	ADM 531.50 EPI 764.25	ADM 540.28 EPI 768.44	60.5 ± 5.07	58.56 ± 3.62	4
	25		22 (77%)		52.52 ± 11.0		ADM 521.14 EPI 770.90		61.00 ± 7.06		

*Exp*, experimental group; *Con*, control group; *BC*, breast cancer; *NHL*, non-Hodgkin lymphoma; *HD*, Hodgkin disease; *ALL*, acute lymphocytic leukemia; *ADM*, adriamycin; *EPI*, epirubicin; *FU*, follow-up; *mon*, months. All the included studies were randomized controlled trials. In the R. S. study, there were two experimental groups receiving different dosages of carvedilol, 12.5 mg and 25 mg once daily

### Effects of carvedilol

#### Asymptomatic LVEF decrease

**Fig. 2:**
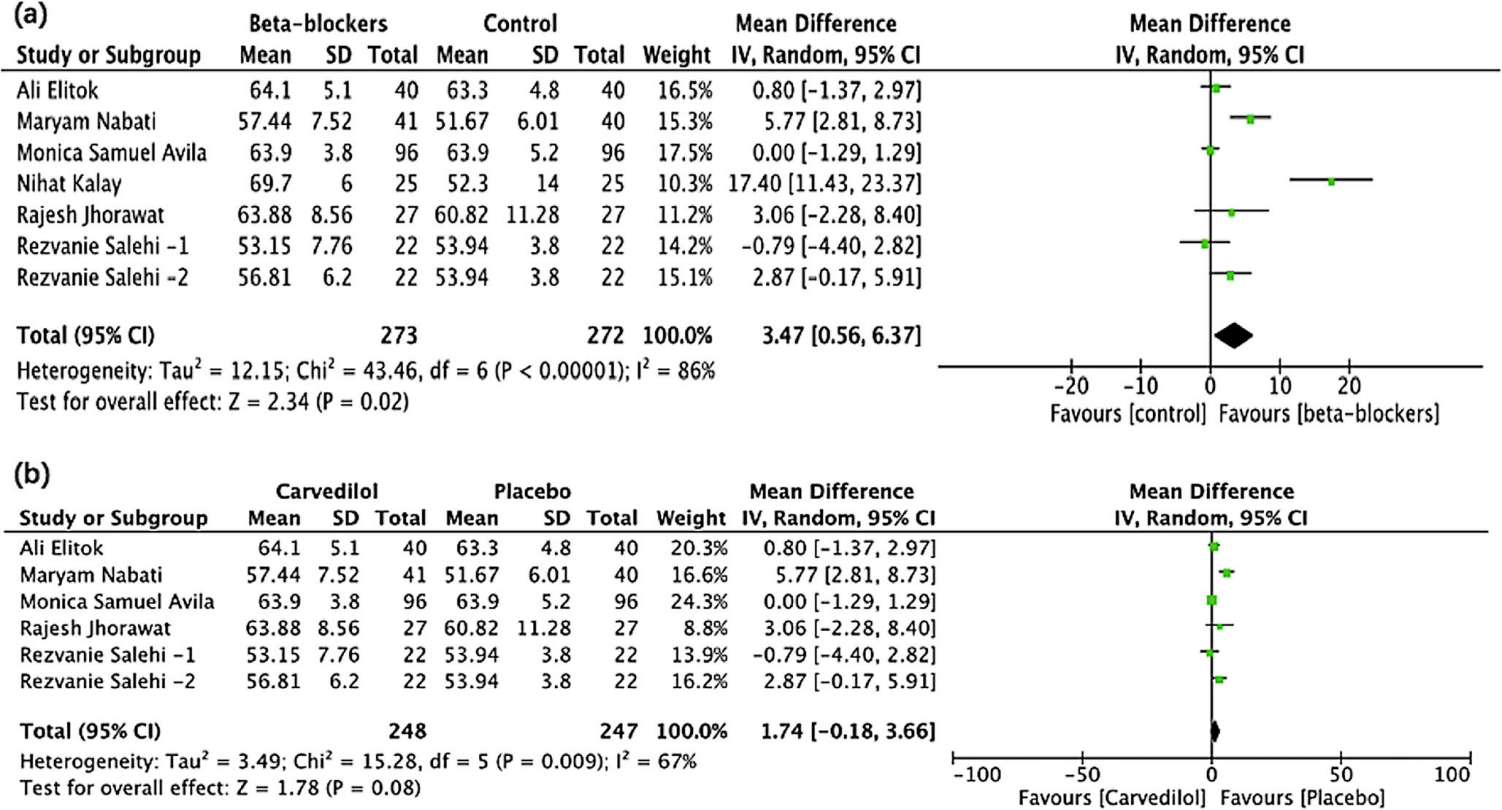
The forest plot of the effect of carvedilol on LVEF post-chemotherapy **a** shows the pooling result of all the studies using carvedilol and **b** shows the pooling result of the studies after the exclusion of the N. K. study. Rezvanie Salehi-1 and Rezvanie Salehi-2 were two different experimental groups in the same study, receiving different dosage of carvedilol, 12.5 mg and 25 mg once daily

Pooling of the LVEF was available in six trials comprising 533 patients (average age, 48.1 years; female patients, 89%). The baseline LVEF between the carvedilol and placebo groups was not significantly different (64.0% vs 64.5%; *P* = 0.52). At the end of the follow-up (4–6 months), the pooling result revealed a statistically significant small difference between the two groups (MD, 3.47; 95% CI, 0.56–6.37; *P* = 0.02). The heterogeneity was substantial (*I*^2^ = 86%) (Fig. 2).

#### Sensitivity analysis of the primary outcome

The Galbraith plot revealed that the substantial heterogeneity came from the two studies by N. K. et al. [24] and M. N. et al. [27]. The exclusion of the study by N. K. et al. reduced *I*^2^ from 86 to 67% (MD, 1.74; 95% CI, − 0.18 to 3.66; *P* = 0.08). Excluding the study by M. N. et al., heterogeneity further decreased from 67 to 8% (MD, 0.51; 95% CI, − 0.47 to 1.09; *P* = 0.31). Then, with any single study excluded from among the rest, the pooling results remained similar and did not differ from the overall estimate (Online Fig. 1 and Fig. 2 in the Supplement).

#### Clinically overt cardiotoxicity and all-cause mortality

**Fig. 3:**
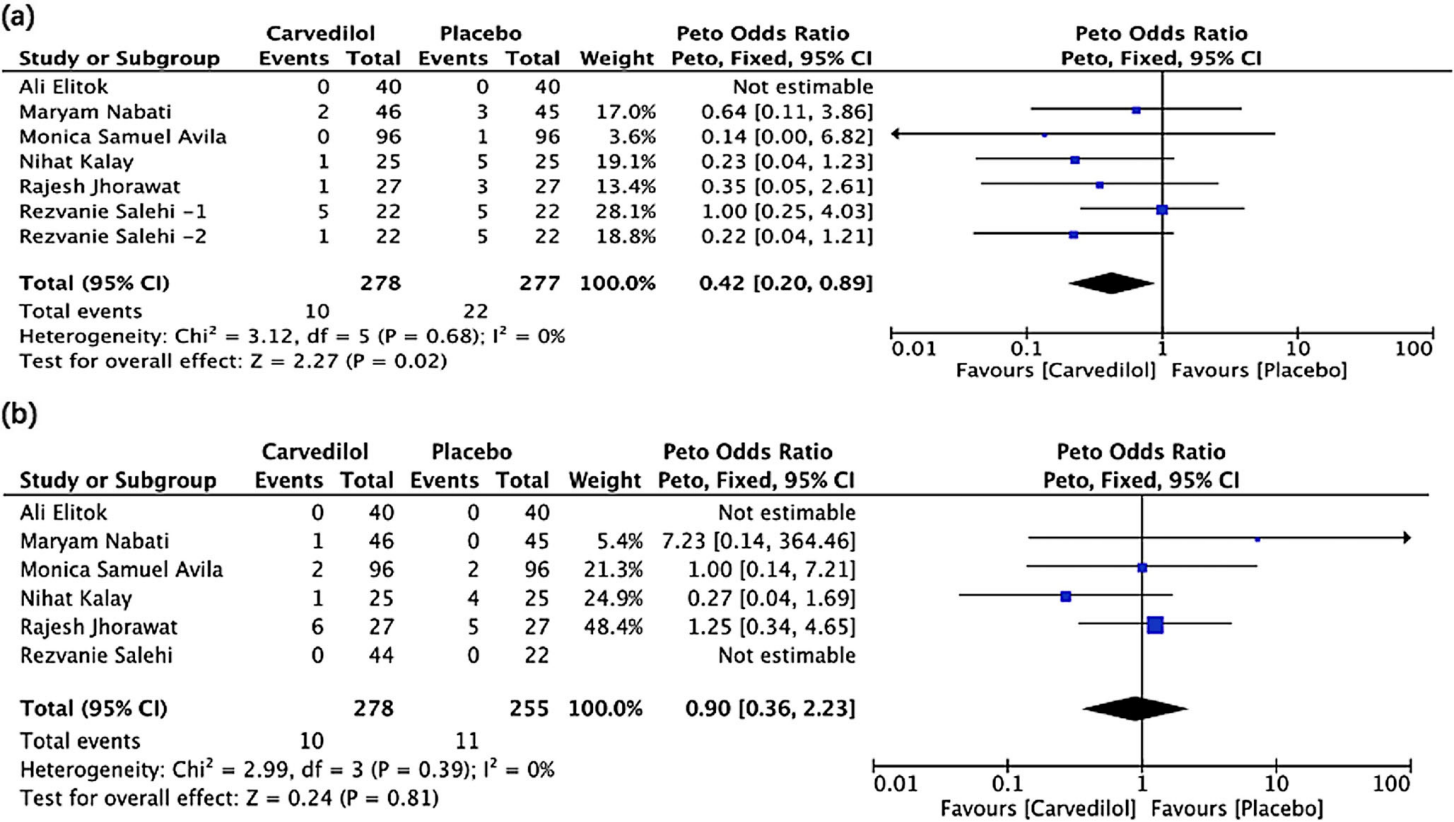
Forrest plot of the effect of carvedilol on clinically overt cardiotoxicity (**a**) and all-cause mortality (**b**). Rezvanie Salehi-1 and Rezvanie Salehi-2 were two different experimental groups in the same study, receiving different dosage of carvedilol, 12.5 mg and 25 mg once daily

The aggregated results revealed that the incidence of clinically overt cardiotoxicity was lower in the carvedilol group than the placebo group (Peto OR, 0.42; 95% CI, 0.20–0.89; *P* = 0.02). In the study by Elitok [26], none of the patients developed overt cardiotoxicity in both groups. For the estimate of all-cause mortality, 10 deaths among 278 patients (3.6%) were reported in the carvedilol group and 11 deaths among 255 patients (4.3%) in the placebo group. The pooled estimate revealed no statistically significant difference in the two groups (Peto OR, 0.90; 95% CI, 0.36–2.23; *P* = 0.81). No case of death was reported in either group in two studies [26, 29] (Table 2 and Fig. 3).

**Table 2:** All-cause mortality and clinically overt cardiotoxicity in studies using carvedilol

Study	All-cause mortality		Clinically overt cardiotoxicity		
	Carvedilol	Placebo	Cutoff point	Carvedilol	Placebo
N. K. [24]	1 (4%)	4 (16%)	LVEF < 50%	1 (4%)	5 (20%)
R.J. [25]	6 (22%)	5 (18%)	LVEF < 50%	1 (4%)	3 (12%)
A. E. [26]	0	0	Interrupted chemotherapy due to cardiotoxicity	0	0
M. N. [27]	1 (2%)	0	LVEF < 40%	2 (4%)	3 (7%)
R. S. [29]	2 (9%) [12.5 mg carvedilol]	4 (18%)	Systolic cardiomyopathy (no specification)	5 (23%) [12.5 mg carvedilol] 1 (5%) [25 mg carvedilol]	5 (23%)
	1 (4%) [25 mg carvedilol]		Diastolic cardiomyopathy (no specification)	3 (14%) [12.5 mg carvedilol] 3 (14%) [25 mg carvedilol]	5 (23%)
M. A. [28]	2 (2%)	2 (2%)	LVEF reduction ≥ 10%	14 (14.5%)	13 (13.5%)
			LVEF decreased to < 55%	0	1 (1%)

The cutoff point of clinically overt cardiotoxicity is based on the criteria used in individual study

### Change of the LV end-diastolic diameter

Pooling of LV end-diastolic diameter was available in three studies. Post-chemotherapy, the LV end-diastolic diameter increased in the placebo group compared with the carvedilol group (MD, − 1.41; 95% CI, − 2.32 to − 0.50; *P* = 0.002). No significant heterogeneity was detected (*I*^2^ = 31%) (Online Fig. 3 in the Supplement).

### Diastolic dysfunction

We extracted the E/A ratio from five trials. The pooled results did not exhibit the statistical significance (MD, 0.03; 95% CI, − 0.03 to 0.09; *P* = 0.29). No significant heterogeneity was detected (*I*^2^ = 27%) (Online Fig. 4 in the Supplement).

### Risk of bias in the included studies

**Fig. 4:**
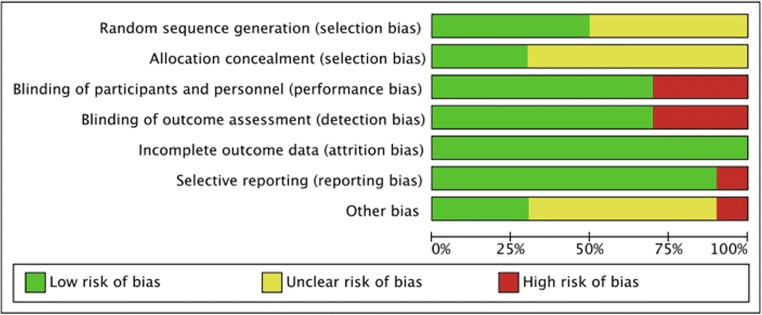
Summary figure of the risk of bias presented to reveal the proportion of studies with each of the judgements (low risk, high risk, and unclear risk)

Both summary and graph figures of the risk of bias were presented to reveal the proportion of studies with each of the judgments (“low risk,” “high risk,” and “unclear risk”) (Fig. 4). Among included studies, while four were conducted with appropriate randomization, others did not describe the methods for randomization, although words such as “randomly” and “randomized” were mentioned. Most articles did not report allocation concealment, except Pituskin [22], Gulati [23], and Avila [28], using central dispensation or sealed envelopes. We classified the three open-labeled studies as “high risk.” M. N. et al. could not follow some patients throughout the study, but the attrition in both groups seemed balanced (experimental group, four patients; control group, five patients). In the study by Avila, eight patients had no valid randomization, thus were excluded in the trial. N. K. et al. neither reported the standard deviation (SD) of the post-chemotherapy LVEF nor replied to our e-mail; thus, we could not eliminate the possibility of “high risk” for selective reporting. Finally, Egger’s test did not exhibit a significant published bias regarding the primary outcome (*P* = 0.127).

## Discussion

We systematically reviewed ten prospective RCTs for four beta-blockers, carvedilol (six studies; 545 participants), nebivolol (one study; 45 participants), metoprolol (two studies; 146 participants), and bisoprolol (one study; 61 participants), and used meta-analysis to quantitatively assess, at least, three studies for the following outcomes: LVEF, LV end-diastolic diameter, E/A ratio, clinically overt cardiac dysfunction, and all-cause mortality. This review could update the information on the effects of the prophylactic use of carvedilol for cardioprotection in the setting of chemotherapy-mediated cardiotoxicity and help with a comprehensive therapy regimen design for cancer survivors.

Chemotherapy-induced cardiotoxicity incorporates multiple cardiovascular manifestations and is mostly characterized by an asymptomatic decline in the LVEF [30]. Our initial outcome corroborated this and exhibited a considerable small LVEF decrease in the placebo group than the carvedilol group. Per the Galbraith plot, the substantial heterogeneity was primarily attributed to two studies, N. K. et al. and M. N. et al. By excluding the study by N. K. et al., the heterogeneity declined to 67%, but the outcome had been reversed (MD, 1.74; 95% CI, − 0.18 to 3.66; *P* = 0.08). Several things could be accountable for this discrepancy. First, patients in this study received relatively higher doses of anthracyclines and, thus, were supposed to exhibit a higher incidence of cardiotoxicity considering the dose-dependent feature of anthracycline [10]. And this could make the effect of using a cardioprotective agent more tangible. However, the contemporary chemotherapy protocol had been reformed, and the anthracycline dosage was much lower in the latest studies. Second, the SDs of the LVEF were not provided initially but extracted from another review [31], which could have affected the results. Third, the sample size was small (25 patients/group). The pooled result of the remaining studies demonstrated that carvedilol exerted no impact on the early asymptomatic LVEF reduction (MD, 1.74; 95% CI, − 0.18 to 3.66; *P* = 0.08; *I*^2^ = 67%). Regarding the heterogeneity from the study by M. N. et al., one possible reason could be that, opposed to most included trials, the average daily dose of carvedilol was much lower (6.7 vs 12.5 mg, q.d.). Although the LVEF was similar at the baseline in the two groups in this study, troponin I was considerably higher in the control group than the carvedilol group, suggesting that the control group could have more impaired myocardium than the carvedilol group at the baseline. To the best of our knowledge, the study by Avila [28] was the latest RCT on this subject with the largest sample size and the most credible results thus far. And our results were more in agreement with this study. In the trial by Elitok [26], although the LVEF remained unchanged in both groups, carvedilol did exhibit a protective effect by preventing the decrease in LV strains, as strain imaging is more sensitive than the LVEF when detecting the subclinical cardiac damage [32].

Our pooled results revealed a lower incidence of overt cardiotoxicity in patients receiving carvedilol than those receiving placebo, strengthening the conclusion that carvedilol could help prevent the deterioration of the cardiac function when previous studies only reported the numerical difference. Admittedly, different cutoff values were adopted among the included studies, but considering that the clinical endpoints of chemotherapy-induced cardiotoxicity remained debatable and the patient population is highly heterogeneous in the practical oncological setting, a safe conclusion could be that the prophylactic use of carvedilol could decrease the incidence of overt cardiac dysfunction in patients with cancer. However, the ultimate effect awaits confirmation by extensive, well-designed clinical trials.

Our analysis did not observe a statistically significant difference between the two groups regarding the 6-month all-cause mortality. Conversely, another meta-analysis [33], which pooled data from five RCTs with 317 patients, reported that beta-blockers correlated with the reduction of all-cause mortality (Peto OR, 0.33; 95% CI, 0.12–0.92; *P* = 0.03) in patients undergoing chemotherapy. However, as the full text of this meta-analysis was unavailable, more detailed documents would be needed to ascertain whether this discrepancy arose because of the types of beta-blockers or the included population. As these studies did not offer specific reasons for deaths, cardiovascular mortality could not be separately assessed in both our reviews.

Pooling LV end-diastolic diameters revealed that carvedilol use could inhibit the enlargement of the LV chamber, suggesting that carvedilol could affect LV remodeling in this setting. The PRADA trial [34] tested whether candesartan and/or metoprolol could prevent anthracycline therapy–associated interstitial fibrosis by T1 mapping and ECV, which was proved to correlate favorably with myocardial biopsy measurements [35].

Regarding the diastolic function, the study by Avila reported a lower incidence of diastolic dysfunction in the carvedilol group than the placebo group (37.2% vs 28.5%; *P* = 0.039). In fact, most studies reported that the E/A ratio reflects the diastolic function in patients. Our aggregated outcome of the E/A ratio did not exhibit a statistical difference in the two groups.

Pooling of the results was impossible for the other three beta-blockers (nebivolol, metoprolol, and bisoprolol) because of the insufficient number of studies. The study by Kaya et al. [36] using nebivolol and that by Pituskin [22] using bisoprolol reported a cardioprotective effect, but not for the two studies using metoprolol [23, 37]; results were summarized in Table 3. The power of the evidence was considered relatively low either because of the small sample sizes or because of inadequate reporting. The results about the effect of metoprolol were consistent in the two studies; both invalidated the use of metoprolol in this scenario. An earlier meta-analysis [38], which pooled results of the three different beta-blockers (nebivolol, metoprolol, and bisoprolol), reported an insignificant difference in the LVEF change (WMD, 3.05; 95% CI, − 7.22 to 1.12; *P* = 0.15) between groups, but a lower heart failure incidence (OR, 0.33; 95% CI, 0.14–0.80; *P* = 0.01) in the beta-blocker group, which was to some extent consistent with our outcomes.

**Table 3:** Characteristics of studies using other beta-blocker

Author year	Drug	Type of cancer	Sample size (female, %)		Age		Cumulative anthracycline doses (mg/m^2^)		Baseline LVEF (%)		Post-chemotherapy LVEF (%)		FU	Adverse events
			Exp	Con	Exp	Con	Exp	Con	Exp	Con	Exp	Con		
M. K. (2012) [36]	Nebivolol	BC	27 (100%)	18 (100%)	51.4 (9.4)	50.5 (11.1)	EPI 361 ADM 257	EPI 348 ADM 235	65 (4.8)	66.6 (5.5)	63.8 (3.9)	57.5 (5.6)	6 m	No HF, hospitalization or death^†^
E. P. (2016) [22]	Bisoprolol	BC	31 (100%)	30 (100%)	53 (10)	51 (7)	592	643	62 (4)	61 (5)	61 (4)	56 (4)	52 w	CTRCD (1 vs 6); interrupted TRZ (3 vs 9)*
P. G. (2010) [37]	Metoprolol	Lymphoma	42 (48%)	40 (47%)	51.0 (18)	49.1 (19.4)	387.5	386.4	67.7 (5.0)	67.6 (7.1)	63.3 (7.4)	66.6 (6.7)	12 m	No death or interruption of CT^†^
G. G. (2016) [23]	Metoprolol	BC	32 (100%)	32 (100%)	50.5 (9.1)	50.8 (9.2)	NA	NA	63.5 (5.0)	63.6 (4.1)	60.8 (4.7)	63.6 (4.1)	10–61 w	No symptomatic HF^†^

*In the E. P. study, CTRCD was less common in the bisoprolol group (1 of 31 patients) post-cycle 4 compared with placebo (6 of 30 patients; *P* = .02), and interruptions of trastuzumab therapy as a result of LV dysfunction were fewer in the bisoprolol group (3 of 31 patients) compared with placebo (9 of 30 patients; *P* = .03)

†For the other three studies, there was no adverse event in either group

*Exp*, experimental group; *Con*, control group; *FU*, follow-up; *RCT*, randomized controlled trials; *EPI*, epirubicin; *ADM*, adriamycin; *TRZ*, trastuzumab; *CT*, chemotherapy; *BC*, breast cancer; *NA*, not available; *HF*, heart failure; *CTRCD*, cancer therapy–related cardiac dysfunction; *m*, months; *w*, weeks. SDs of age, baseline LVEF, and post-chemotherapy LVEF are expressed in the parenthesis

## Limitations and potential biases in the review process

First, only adult patients were included in this review. We excluded young patients in this study because of some differences between adult and pediatric patients. However, admittedly, childhood cancer survivors are another large group affected by chemotherapy-related cardiotoxicity, which we intend to address in a future study. Second, our conclusions remained generalized to the long-term setting, as the follow-up duration of all the included trials ended at 4–6 months. Finally, only the effects of carvedilol were quantitatively assessed. Hence, further clinical trials are warranted for the estimation of other beta-blockers such as nebivolol, bisoprolol, and metoprolol.

## Conclusions

The prophylactic use of carvedilol exerts no impact on the early asymptomatic LVEF decrease but seemingly attenuates the frequency of clinically overt cardiotoxicity and prevents ventricular remodeling. Nevertheless, prolonged and extensive studies are warranted to validate the efficacy of carvedilol.

## Electronic supplementary material

ESM 1 (PDF 286 kb)
