# Supplementary material for: Protective role of beta-blockers in chemotherapy-induced cardiotoxicity—a systematic review and meta-analysis of carvedilol
Source: Heart Fail Rev. 2018 Dec 6;24(3):325–33. doi: 10.1007/s10741-018-9755-3 (PMC6476829; doi:10.1007/s10741-018-9755-3)

Online supplementary materials

Protective role of beta-blockers in chemotherapy-induced cardiotoxicity——A systematic review and meta-analysis of carvedilol

Journal: *Heart failure reviews*

Shan Huang[1,2#], MS • Qin Zhao[2#], MD • Zhi-gang Yang[2†], MD • Kai-yue Diao[2], MD • Yong He[3], MD • Ke Shi[2], MD • Meng-ting Shen[2], MS • Hang Fu[1], MS • Ying-kun Guo[1†], MD

1. Department of Radiology, Key Laboratory of Birth Defects and Related Diseases of Women and Children of Ministry of Education, West China Second University Hospital, Sichuan University, China

2. Department of Radiology, West China Hospital, Sichuan University, 37# Guo Xue Xiang, Chengdu, Sichuan 610041, China

3. Department of Cardiology, West China Hospital, Sichuan University, 37# Guo Xue Xiang, Chengdu, 610041, China

† Corresponding author:

Zhigang Yang, PhD, MD

Department of Radiology, State Key Laboratory of Biotherapy, West China Hospital, Sichuan University, No.37 Guoxue Xiang, Chengdu, 610041, China

Tel: +86-28-85423817(O)

E-mail: yangzg666@163.com

Yingkun Guo, MD

Department of Radiology, Key Laboratory of Obstetric & Gynecologic and Pediatric Diseases and Birth Defects of Ministry of Education; West China Second University Hospital, Sichuan University, 20# South Renmin Road, Chengdu, Sichuan 610041, China.

Phone No: +86-18980006572

Fax No: +86 28-85502946(H)

Email Address: gykpanda@163.com

Online Fig. 1 Galbraith plot regarding the primary outcome (LVEF post chemotherapy)

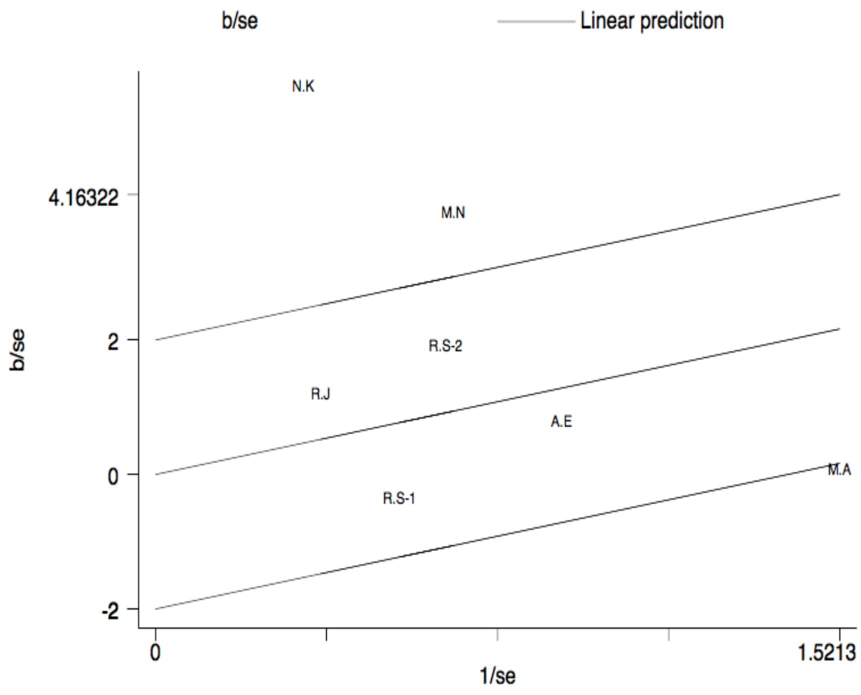

The Galbraith plot constructed on Stata software shows that the M.N. study and N.K. study were the main source of heterogeneity.

Online Fig. 2 Sensitivity analysis regarding the primary outcome (LVEF post chemotherapy)

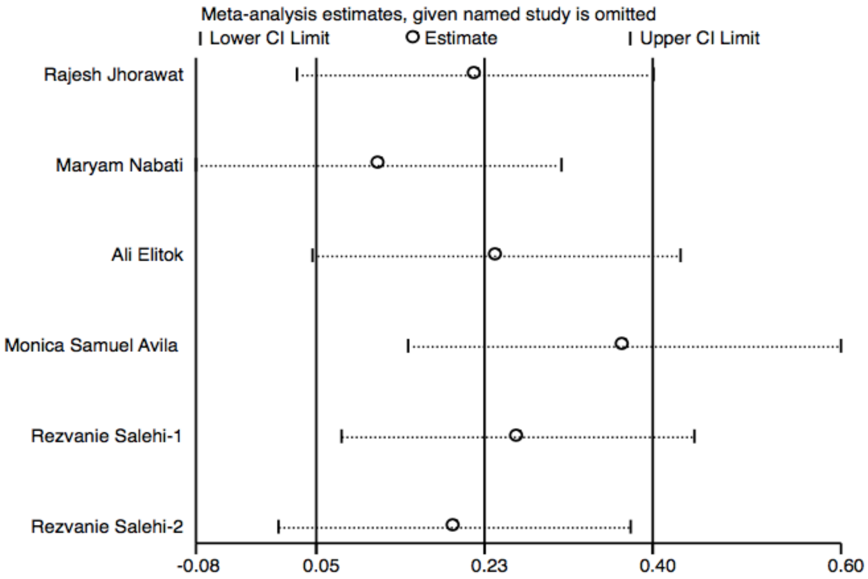

The sensitivity analysis showed that, with any single study excluded from the studies above, the pooling results remained similar and did not differ from the overall estimate.

Online Fig. 3 Forrest plot of LV-end diastolic diameter in carvedilol and control group

	Carvedilol			Placebo				Mean Difference
Study or Subgroup	Mean	SD	Total	Mean	SD	Total	Weight	IV, Fixed, 95% CI
Monica Samuel Avila	45.2	3.2	96	46.4	4	96	78.6%	-1.20 [-2.22, -0.18]
Nihat Kalay	47.4	3.7	25	50.9	5.6	25	11.9%	-3.50 [-6.13, -0.87]
Rajesh Jhorawat	47.95	5.28	27	48.5	5.75	27	9.5%	-0.55 [-3.49, 2.39]
**Total (95% CI)**			**148**			**148**	**100.0%**	**-1.41 [-2.32, -0.50]**

Heterogeneity: $Chi^2 = 2.91$, df = 2 (P = 0.23); $I^2 = 31\%$
Test for overall effect: Z = 3.05 (P = 0.002)

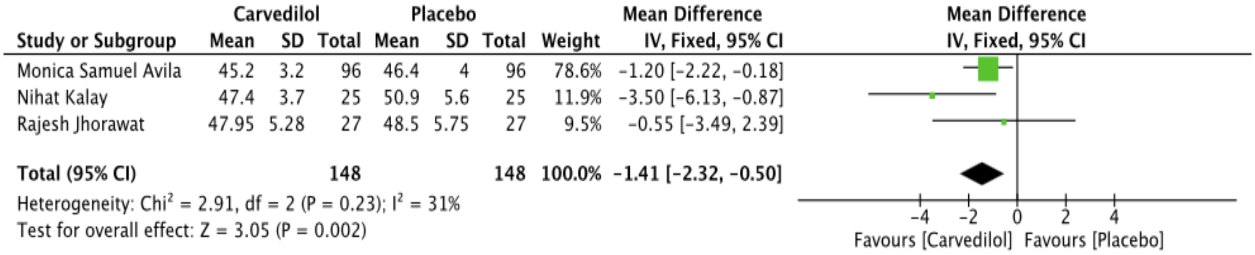

Online Fig. 4 Forrest plot of E/A ratio in carvedilol and control group

Study or Subgroup	Carvedilol			Placebo			Weight	Mean Difference
	Mean	SD	Total	Mean	SD	Total		IV, Fixed, 95% CI
Ali Elitok	1.17	0.3	40	1.09	0.4	40	15.1%	0.08 [−0.07, 0.23]
Maryam Nabati	0.92	0.25	41	0.96	0.28	40	27.0%	−0.04 [−0.16, 0.08]
Nihat Kalay	0.98	0.2	25	0.87	0.2	25	29.4%	0.11 [−0.00, 0.22]
Rajesh Jhorawat	1.18	0.46	27	1.28	0.52	27	5.3%	−0.10 [−0.36, 0.16]
Rezvanie Salehi -1	1	0.38	22	0.86	0.33	22	8.2%	0.14 [−0.07, 0.35]
Rezvanie Salehi -2	0.81	0.17	22	0.86	0.33	22	15.0%	−0.05 [−0.21, 0.11]
**Total (95% CI)**			**177**			**176**	**100.0%**	**0.03 [−0.03, 0.09]**

Heterogeneity: $Chi^2 = 6.82$, df = 5 (P = 0.23); $I^2 = 27\%$
Test for overall effect: Z = 1.05 (P = 0.29)

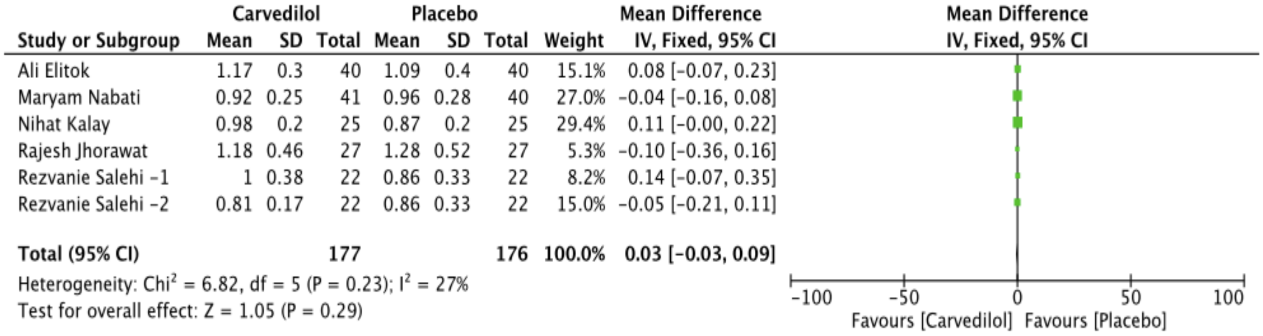

Supplement: Supplementary file 1 — (PDF 286 kb) [file 10741_2018_9755_MOESM1_ESM.pdf]
